# Supplementary figures and images for: Formation of Regulatory Modules by Local Sequence Duplication
Source: PLoS Comput Biol. 2011 Oct 6;7(10):e1002167. doi: 10.1371/journal.pcbi.1002167 (PMC3188502; doi:10.1371/journal.pcbi.1002167)

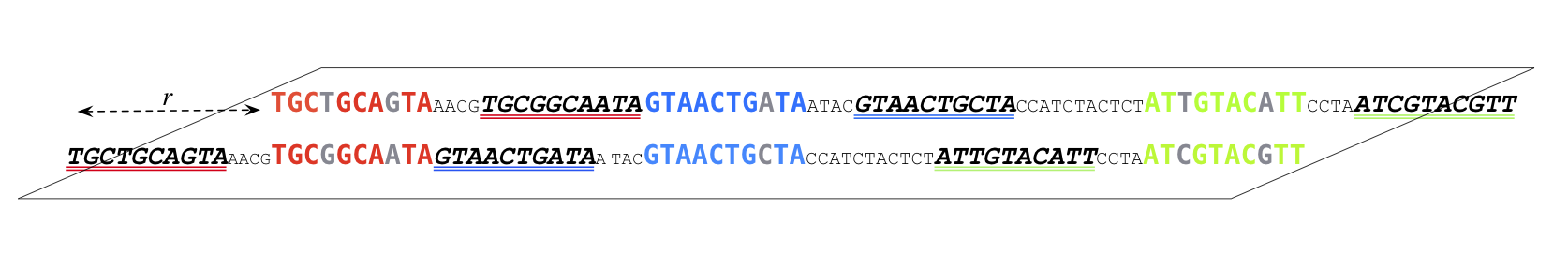

Supplement: Figure S1 — Motif detection in sequence segments (schematic). The figure shows a configuration of correlated sequence sites of length bp and distance bp from each other. Pairs of correlated sites have the following properties: (i) The average mutual similarity between aligned nucleotides is larger than a given threshold, . (ii) The left sites (and, hence, also the right sites) of all pairs have no common nucleotides. This condition is necessary in order to avoid overcounting of mutual similarity in overlapping site pairs. (iii) The sum of the mutual similarities of all pairs in the set is maximal. In the example shown, there are three different motifs with reoccurring sequence patterns marked by different colors (red, blue, green). To illustrate the alignment of the site pairs, we shift the whole sequence by bp in the second row. The left and right site of each motif are shown in boldface in the first and the second row, respectively. Mismatches between aligned sites of the same motif are shown in boldface gray letters. The flanking regions separating the correlated sequence pairs are shown in smaller font. (TIFF) [file pcbi.1002167.s001.tif]
